# Molecular Analysis of the Cyanobacterial Community in Gastric Contents of Egrets with Symptoms of Steatitis

**DOI:** 10.2174/1874285801509010160

**Published:** 2015-11-03

**Authors:** Tomoyasu Nishizawa, Yasuko Neagari, Takamasa Miura, Munehiko Asayama, Koichi Murata, Ken-Ichi Harada, Makoto Shirai

**Affiliations:** 1 College of Agriculture, Ibaraki University, Ibaraki 300-0393, Japan; 2 Laboratory for Intellectual Fundamentals for Environmental Studies, National Institute for Environmental Studies, Ibaraki 305-8506, Japan; 3 College of Bioresource Sciences, Nihon University, Kanagawa 252-0880, Japan;; 4 Graduate School of Environmental and Human Science and Faculty of Pharmacy, Meijo University, Aichi 468-8503, Japan;

**Keywords:** Agricultural reservoir, cyanobacterial community, gastric content, Microcystis, microcystin biosynthesis (mcy) gene, T-RFLP profiling.

## Abstract

Many deaths of wild birds that have drunk water contaminated with hepatotoxic microcystin-producing cyanobacteria have been reported. A mass death of egrets and herons with steatitis were found at the agricultural reservoir occurring cyanobacterial waterblooms. This study aimed to verify a hypothesis that the egrets and herons which died in the reservoir drink microcystin-producing cyanobacteria and microcystin involves in the cause of death as well as the symptoms of steatitis. The cyanobacterial community in gastric contents of egrets and herons that died from steatitis was assessed using cyanobacterial 16S rRNA-based terminal-restriction fragment length polymorphism (T-RFLP) profiling and a cyanobacterial 16S rRNA-based clone library analysis. In addition, PCR amplification of the mcyB–C region and the mcyG gene, involved in microcystin biosynthesis, was examined. The cyanobacterial community in the gastric contents of two birds showed a simplistic composition. A comparison of cyanobacterial T-RFLP profiling and cloned sequences suggested that the genus Microcystis predominated in both samples of egrets died. Although we confirmed that two egrets which died in the reservoir have taken in cyanobacterial waterblooms containing the genus Microcystis, no mcy gene was detected in both samples according to the mcy gene-based PCR analysis. This study is the first to show the profiling and traceability of a cyanobacterial community in the gastric contents of wild birds by molecular analysis. Additionally, we consider causing symptoms of steatitis in the dead egrets.

## INTRODUCTION

There has been increased concern over the effects on wildlife of toxins, particularly those associated with harmful algal blooms in water reservoirs and recreational areas. Cyanobacteria, which include the genera *Anabaena,* *Microcystis, Nostoc, *and* Planktothrix*, are known as producers of hepatotoxic microcystins [1, 2], the toxicity of which is due to the inhibition of protein phosphatase 1 and 2A [3, 4]. Microcystins acutely cause cytokeratin hyperphosphorylation which leads to a disruption of cytoskeletal components and cell deformation, followed by disruption of the liver architecture [5]. To date, the *mcy* gene encoding microcystin synthetase was characterized from the planktonic unicellular *Microcystis* [6-8], the filamentous *Planktothrix* [9], and the filamentous and heterocystic *Anabaena* [10]. Microcystins are produced by a large multisubunit enzyme complex comprising nonribosomal peptide synthetases (NRPSs) and polyketide synthases (PKSs). 

The deaths of over 50 hemodialysis patients in Caruaru, Brazil, were attributed to exposure to microcystins in the dialysis solution [11]. This type of toxin was responsible for the deaths of wild birds that drunk from a pond with cyanobacterial waterblooms in Hyogo Prefecture, Japan [12]. In the San Diego area, USA, death of Great Blue Herons (*Ardea herodias*) and Black-crowned Night Herons (*Nycticorax nycticorax*) caused steatitis, an inflammation of fat tissue, has been reported from 1979 [13]. Also, the deaths of herons from steatitis was observed in Chesapeake Bay, USA, where toxic cyanobacterial waterblooms containing *Anabaena* spp. occurred [14]. Hypotheses concerning the cause of steatitis include consumption of food high in polyunsaturated fats and intake of the toxic waterbloom, hepatotoxic microcystin [13, 14]. Microcystins were detected in water samples and in tissue samples from the dead herons and suggested to be a possible cause of the death as well as the symptoms of steatitis [14]. In a similar report, more than 70 egrets and herons were found sick or dead at an agricultural reservoir in Kanagawa Prefecture, Japan [15]. It was confirmed that these birds also died from steatitis. High counts of cyanobacteria were found in the reservoir, and the genera *Microcystis*, *Raphidiopsis* and *Planktothrix* were identified from cell and colony morphology. However, no microcystin was detected in the reservoir’s water or the livers of the dead birds [15]. To verify that toxic cyanobacteria is responsible for the cause of death, it is necessary to reveal whether egrets and herons which died in the reservoir drunk the cyanobacterial waterblooms containing microcystin-producer.

Molecular methods such as 16S rRNA-based terminal restriction fragment length polymorphism (T-RFLP) [16, 17] have been utilized to assess the dominant microbes in environmental samples using an automated DNA sequencer with quenching-fluorescence-labeling fragments [18-20]. T-RFLP profiling has become a valuable technique in microbial ecology and can be performed quickly and easily with a high resolution that allows even related strains of bacteria to be differentiated [21-25]. The genetic characterization of cyanobacterial strains has been performed using a bacterial 16S rRNA-based amplified rDNA restriction analysis (ARDRA) [26]. Generally, the 16S rRNA sequence or 16S-23S rRNA internal transcribed spacer sequence (16S-23S ITS) is utilized to classify *Microcystis* species [27]. Additionally, Nübel *et al.* [28] provided cyanobacterial specific primers for the selective retrieval of cyanobacterial ARDRA data from an environmental sample. The cyanobacterial primers were used for the identification, typing, and monitoring of cyanobacteria in freshwater reservoirs [29].

The objective of this study was to characterize the cyanobacterial community in the gastric contents of dead egrets. To assess the cyanobacterial community structure, cyanobacterial 16S rRNA-based T-RFLP profiling as well as a clone library analysis was performed. Moreover, to examine whether toxic cyanobacteria exist in the gastric contents, PCR amplification of the *mcy* gene was carried out.

## MATERIALS AND METHODS

### Collection of Samples and Cyanobacterial Strains

The agricultural reservoir was located in Yokosuka, Kanagawa Prefecture, Japan (35(12’ N, 139(37’ E). Many egrets and herons were died in biotope which is installed in the center of the reservoir. Deep frozen gastric contents of egrets and herons were provided by the National Institute for Environmental Studies (NIES, Tsukuba, Japan) (Table **1**). Six samples of the gastric contents were stored at -80^o^C until DNA was extracted. *Microcystis aeruginosa* NIES-87 was obtained from NIES. *M. aeruginosa *strains B-19 and K-139 were isolated from Lake Kasumigaura in Ibaraki Prefecture, Japan [6]. Strains were grown in CB medium at 30^o^C with continuous illumination under fluorescent (cool white) lights (35 µmol/m^2^/s) [30].

### DNA Extraction and Manipulation

Total cyanobacterial DNA was isolated from cells grown to the late logarithmic phase using a previously described procedure [6]. The samples of gastric content (0.5 g wet weight) cryopreserved for one year and a half were washed with TES buffer (0.05 M Tris-HC1, 0.1 M NaC1, 0.05 M EDTA; pH 8.0) and resuspended in 0.5 ml of SET buffer (0.05 M Tris-HCl, 0.05 M EDTA, 25% [wt/vol] sucrose; pH 8.0). Lysozyme treatment was performed on ice for 5 hours. Total DNA was isolated as described previously [6]. 

### Amplification of the Bacterial and Cyanobacterial 16S rRNA Genes

The bacterial 16S rRNA gene in extracted DNA (0.1 µg) was amplified using the forward primer bac10F and reverse primer bac1492R [20], and PCR amplification was performed as described previously [20]. The cyanobacterial 16S rRNA gene in extracted DNA (0.1 µg) was amplified using the forward primer CYA106f (5’-CGGACGGGTGAG TAACGCGTGA-3’) [28] and reverse primer CYA792r (5’-TCCCCTAGCTTTCGTCCC-3’) following the MiCA3 program [31]. The reaction was performed in a Takara PCR Thermal Cycler Personal (Takara Bio, Otsu, Japan) with Takara Ex *Taq* polymerase (Takara Bio) under the following conditions: 2 min at 95^o^C, followed by a cycle of 95^o^C (30 s), 54^o^C (45 s), and 72^o^C (90 s) and a final extension for 5 min at 72^o^C. To avoid PCR bias [32] and the heteroduplex-form of 16S rRNA [33], the number of amplification cycles was fixed at twenty-two in KS2P and KS4P samples. The cyanobacterial 16S rRNA gene PCR amplicon was observed at approximately 650-bp on the agarose gels. To construct the clone library, the amplicon of the cyanobacterial 16S rRNA gene region was ligated into the pGEM-T-easy cloning vector (Promega, Madison, WI) and ligation products were transformed into *Escherichia coli* DH5aMCR (Cosmo Bio, Tokyo, Japan) as described previously [18]. PCR products were resolved on 1% agarose gels in Tris-Boreate-EDTA (TBE) buffer.

### T-RFLP Profiling

The cyanobacterial 16S rRNA gene in the extracted DNA was amplified using CYA106f (see the above section for the nucleotide sequence) and QCYA792r (5’-CTCCCCTAGCT TTCGTCCC-3’). The 5’-end fluorescence-labeled primer [34], QCYA792r, was purchased from Nippon Steel & Sumikin Eco-Tech Crop., Ltd. (Tsukuba, Japan). The PCR mixture (30 µl) was prepared by combining 0.1-0.01 µg of template-extracted DNA, 1.0 µl of 10 pmol/µl primers, Takara Ex *Taq* polymerase, dNTPs, and 3.0 µl of optimized 10-fold Ex buffer (Takara Bio) in a PCR thermal cycler. The PCR of the 16S rRNA gene for T-RFLP profiling was carried out under the following conditions: 1 min at 95^o^C, followed by 95^o^C (30 s), 54^o^C (45 s), and 65^o^C (90 s). 

The PCR-based DNA fragment analysis by T-RFLP was performed as described previously [35]. The purified T-RF DNA was mixed with 15 µl of Hi-Di formamide and 0.1 µl of DNA standard LIZ^®^ 500 (Applied Biosystems) for standardization. The precise lengths of terminal restriction fragments (T-RFs) from the amplified fragments were determined on PE Applied Biosystems Automated DNA Sequencer (model 3130*xl*) (Applied Biosystems, Foster, CA). The lengths of fluorescently labeled T-RFs were determined by comparison with internal standards using GeneMapper (version 3.7) software (Applied Biosystems). T-RFLP profiling of the cyanobacterial community structure in samples were obtained with peaks ranging from 0 to 700 bases. The definition of the T-RFs were peaks with a fluorescence threshold of more than 30.

### DNA Sequencing and Computer Analysis

Nucleotide sequences were determined with 3130*xl* DNA sequencer (Applied Biosystems) and DNA sequencing reactions were carried out using a BigDye™ Terminator Cycle Sequencing Ready Reaction Kit (Applied Biosystems) according to the protocol of the manufacturer. The double-stranded DNA sequences were assembled and analyzed using Genetyx (version 11.0) and Genetyx-ATSQ (version 4.0) software (Genetyx Co., Tokyo, Japan), respectively. Similarity to DNA sequences retrieved from the DDBJ/EMBL/GenBank databases using BLAST.

### PCR Amplification of the *mcyG* Gene and Non-Coding Region

To amplify the *mcyG* gene, which is involved in the biosynthesis of Adda (3-amino-9-methoxy-2,6,8-trimethyl-10-phenyldeca-4,6-dienoic acid) in microcystin [7], the primers 5’-McyG12AT and 3’-McyG12AT [36] and 0.1-0.01 µg of template-extracted DNA were used. The reaction was performed in a PCR thermal cycler. For the amplification, Takara LA *Taq* polymerase (Takara Bio) was used. The reaction was performed under the following conditions: 3 min at 95^o^C, followed by 5 cycles of 95^o^C (30 s), 54^o^C (45 s), and 72^o^C (90 s), and then 30 cycles of 95^o^C (30 s), 60^o^C (30 s), and 72^o^C (90 s) and a final extension for 3 min at 72^o^C. To generate the amplicon between the *mcyB* and *mcyC* genes, primers sets were used as described previously [6, 37]. To generate the amplicon of the non-coding region between the *dnaN* and *uma1* genes and between the *hypX* and *uma1* genes, primers set were used and the *dnaN*-*uma1* region and the *hypX*-*uma1* region were amplified as described previously [37]. For positive controls of PCR amplification, microcystin-producing *M. aeruginosa* K-139, and non-*mcy*-possessing *M.* *aeruginosa* strains, B-19 (for amplification of the *dnaN*-*uma1* region) and NIES-87 (for the *hypX*-*uma1* region), were used. These PCR products were resolved on 1% and 1.5% agarose gels in TBE buffer.

### Sequence Accession Numbers

All cloned sequences in this study were deposited in the DNA Database of Japan (DDBJ) under accession numbers AB689760 to AB689772.

## RESULTS AND DISCUSSION

As the gastric contents of egrets and herons died have been cryopreserved in the NIES, molecular analysis may reveal the existence of toxic cyanobacteria. In this study, we succeeded in extracting gastric content DNA from two (KS2P and KS4P) of six deep frozen samples (Table **1**). The PCR amplification for the bacterial 16S rRNA gene was confirmed in the KS2P and KS4P samples (Table **1**). In the KS1P, KS3P, KS5P, and KS6P samples, there were almost no gastric contents since it became weak and was not able to have diets [15]. In addition, quite slightly DNA extracted was fragmented finely and PCR amplification was impossible. The DNA samples, KS2P and KS4P, were used for subsequent analyses.

**Fig. (1):**
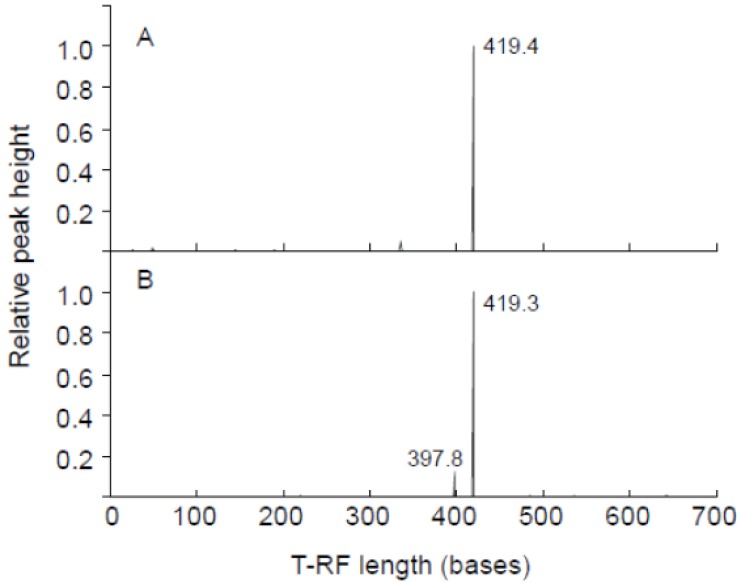
T-RFLP profiling of the cyanobacterial 16S rRNA gene amplified as template total DNA of the gastric content of dead egrets. KS2P (A) and KS4P (B) were used for PCR amplification. HaeIII was used for digestion. The x-axis indicates the terminal restriction fragment length (bases) between 0 and 700 bases, and the y-axis represents the relative height of the peak. The highest peak was calculated as 1. A relative height of over 0.1 showed the T-RF length.

To examine the principle structure of the cyanobacterial community in the DNA samples of gastric content, 16S rRNA-based T-RFLP profiling was performed. A relative peak height of over 0.1 was observed in *Hae*III-digested T-RFLP profiling (Fig. **1**): 419.4-base (KS2P) and 419.3-base (KS4P) experimental T-RFs (T-RFs_exp_) were mainly detected. The cyanobacterial community in both the gastric contents of the birds had a simplistic composition. To identify the dominant T-RF_exp_, a cyanobacterial 16S rRNA-based clone library of gastric content was analyzed. A total of 13 clones were sequenced and taxonomic classification was carried out with the BLAST tool of the NCBI-nr database. Most of the cloned sequences from gastric content were closely related to the genus *Microcystis*, while clone KS2-4 was classified into the genus *Anabaena* (Table **2**). The sequence of clone KS2-7 was identical with those of KS4-7 and KS4-18, suggesting that the two egrets have taken the same diets containing *Microcystis*. On the other hand, the *in silico* T-RF of the cyanobacterial 16S rRNA gene’s cloned sequence (T-RF_seq_) was also used for comparison as shown in Table **2**. The dominant *Hae*III-digested T-RF_exp_s of KS2P and KS4P were mostly consistent with the *Hae*III 421-base T-RF_seq_ of those clones, respectively, except for clone KS2-4. These results indicated that *Microcystis* is the dominant genus in the gastric contents of the egrets died. The wild birds had been seen drinking water from the reservoir where the planktonic *Microcystis*-blooms were observed [15], indicating that the two egrets died may drink the reservoir’s water.

**Fig. (2):**
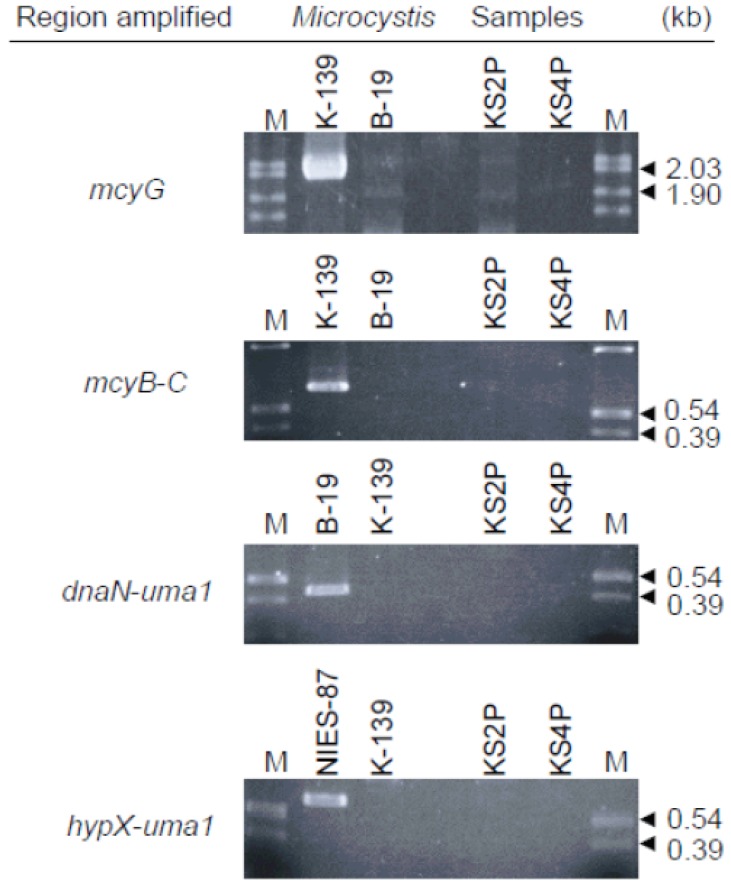
Detection of PCR amplicons by agarose gel electrophoresis. M, Marker; K-139, M. aeruginosa K-139; B-19, M. aeruginosa B-19; NIES-87, M. aeruginosa NIES-87.

The deaths of wild herons in an area of Chesapeake Bay containing harmful algal blooms and toxic cyanobacteria suggested the involvement of a cyanobacterial toxin (microcystin) [14]. Cyanobacterial waterblooms were observed at the surface of the agricultural reservoir where egrets and herons died in Sep. and Oct. 2008, but microcystin was not detected by instrumental analysis (high-performance liquid chromatography, HPLC) from the reservoir’s water on Oct. 2008 and egrets and herons died [15]. To date, no toxic strains of the genus *Microcystis* have been investigated in relation to the microcystin biosynthesis (*mcy*) gene. Although a highly conserved gene organization of the* mcyABC* and *mcyDEFGHIJ* operons in microcystin-producing *Microcystis* strains were revealed [36-38], no correlation between possession of the *mcy* gene and the phylogeny of 16S-23S ITS in domestic *Microcystis* isolates was observed [36, 37]. It was also found that the *mcy* gene cluster invariably is arranged between *dnaN* and *uma1* in *mcy*-possessing *Microcystis, *whereas *dnaN*–*uma1* and *hypX*–*uma1* are aligned in non-*mcy*-possessing *Microcystis* [37]. Therefore, PCR amplification of *mcy* was performed using the DNA samples KS2P and KS4P in order to verify the presence of *mcy*-possessing *Microcystis*. The region amplified was selected so as to target the region between the 3’-end of the *mcyB* gene and 5’-end of the *mcyC* gene (*mcyB*-*C*) [6] and the NRPS module of *mcyG*, which is involved in the biosynthesis of a starter unit, Adda (3-amino-9-methoxy-2,6,8-trimethyl-10-phenyldeca-4,6-dienoic acid), in microcystin [7]. In contrast, to detect microcystin non-producing *Microcystis*, amplification of the non-coding region between *dnaN* and *uma1* and between *hypX* and *uma1* was conducted as described by Noguchi *et al.* [36]. According to the PCR analyses of these regions in each gene, no PCR amplicon of *mcyG*, *mcyB*-*C* region, and its non-cording region was observed in KS2P and KS4P (Fig. **2**), indicating that an unknown non-*mcy*-possessing *Microcystis* was present in the gastric content of the egrets which died.

Sudden appearance of toxic cyanobacteria breaks out in waterbodies. Dynamics of toxic *Microcystis* population in lake observed temporal changes on the seasonal variability [39]. There has been no report that the wild birds died in the pond in Nishinomiya, Hyogo prefecture, since 1995. Similarly, no deaths of wild birds have been reported since 2008 even though cyanobacterial waterblooms consisting mainly of *Microcystis* were observed in the reservoir. Many deaths of egrets and herons which causes with the incidence of steatitis have been reported in USA to date. It was suggested that oxidized polyunsaturated fatty acids and toxic cyanobacteria are a possible cause of steatitis [14, 40]. Rattner and McGowan [14] reported that microcystins were detected in water samples in Chesapeake Bay and in tissue samples of dead herons, and speculated that microcystins were responsible for the death from steatitis of wild birds. However, microcystins have not yet been proved to cause steatitis. Neagari *et al.* [15] reported that no common food items affected with steatitis find in dead egrets and herons in the reservoir because their stomachs were mostly empty, and observed that focal necroses found in liver sections of egret with steatitis. Acute toxic deaths occurred within 3 hours when a fatal dose of microcystin were intraperitoneally (i.p.) injected into mouse [41, 42]. The liver of the mouse which died hypertrophied by bleeding, and a necrosis of the liver has occurred with disruption of the architecture of the hepatic cord. Yoshida *et al.* [43] reported that median lethal dose (LD_50_) of the orally given microcystin estimated is 167 times higher than the i.p. LD_50_ value. Orally administrated microcystin caused primarily hepatocellular injuries with necrosis [43]. Therefore, it is seemed that microcystin is a possible cause of necroses in the egret with steatitis. Pathologic finding of liver sections in the egret died, does not indicate the acute toxicity by microcystin. To our knowledge, no chronic toxicity by microcystin has been reported. Moreover, microcystins inhibit the protein phosphatase 1 and 2A and act as tumor promoters in human liver [4, 44]. Extracted microcystin-producing *Microcystis* cells are capable of eliciting a production of interleukin-1 (IL-1) [41] and tumor necrosis factor alpha (TNF-a) [42]. Additionally, a delayed hypersensitivity reaction occurs by extracted non-toxic *Microcystis* cells [45]. *Microcystis* produce small peptides containing novel non-protein amino acids are synthesized by NRPS: the NRPS gene clusters are responsible for the biosynthesis of protease inhibitor micropeptins [46] and aeruginosin [47] in the genus *Microcystis* were confirmed using the gene knockout. NRPS and PKS genes of unknown function have been identified in *Microcystis *as well [19, 48]. Furthermore, cyanobacteria, including microcystin-producing strains, produce a large number of peptide compounds with varying bioactivities [49, 50]. The filamentous cyanobacterium *Cylindrospermopsis *produces hepatotoxin (i.e. cylindrospermopsin) [51, 52], which also inhibits pyrimidine synthesis [53]. Some unknown product of cyanobacteria other than microcystins may have been a cause of the symptoms of steatitis. In addition, we expected that some pesticide residues are concerned with steatitis. As a result of investigating 329 kinds of pesticide residues in an egret which died in the reservoir on Oct. 2008, no pesticide residue was detected to date (Y. Neagari unpublished data).

## CONCLUSION

Our results revealed that the dead egrets took in water containing cyanobacterium *Microcystis* and showed that microcystin was not associated directly with the death of at least two wild egrets. To our knowledge, this study is the first to show the profiling and traceability of a cyanobacterial community in the gastric contents of wild birds with symptoms of steatitis using a molecular analysis. Although necrosis which may be caused by hepatotoxin was observed in the dead egret’s liver [15], the association between exposures to other bioactive compounds produced by cyanobacteria and the symptoms of steatitis needs to be elucidated in order to verify the cause of death.

## Figures and Tables

**Table 1.** Summary of DNA extraction and PCR amplification from gastric contents.

Sample name	Sampling data	Species	DNA extraction	PCR amplification*a*
KS1P	11 Oct. 2008	*Mesophoyx intermedia*	n.a.	n.t.
KS2P	11 Oct. 2008	*Ardea alba*	+	+
KS3P	13 Oct. 2008	*M. intermedia*	n.a.	n.t.
KS4P	19 Oct. 2008	*A. alba*	+	+
KS5P	19 Oct. 2008	*Nyticorax nycticorax*	n.a.	n.t.
KS6P	19 Oct. 2008	*N. nycticorax*	n.a.	n.t.

^a^ Bacterial 16S rRNA gene region was amplified.

n.a., not available.

n.t., not tested.

+, positive.

**Table 2.** Detected T-RF sizes based on cyanobacterial T-RFLP profiling and in silico T-RF of the cyanobacterial 16S rRNA gene’s cloned sequence.

Method*a*	Sample/Clone	Length (bp)	*Hae*III-T-RFs (bp)	Identity (%)	Closest sequence [Accession number]
T-RFLP	KS2P		419.4		
	KS4P		419.3		
C.L.	KS2-1	634	421	99	*M. aeruginosa *LMECYA 188 [EU078506]
	KS2-4	635	33	98	*A. circinalis* LMECYA 123 [EU078519]
	KS2-6	634	421	99	*M. aeruginosa* LMECYA 91B [EU078497]
	KS2-7, KS4-7, 18	634	421	99	*M. aeruginosa* LMECYA 110 [EU078499]
	KS2-10, KS4-8	634	421	99	*M. aeruginosa* LMECYA 151 [EU078502]
	KS2-12	634	421	99	*M. aeruginosa* LMECYA 29 [EU078489]
	KS4-1	634	421	99	*M. novacekii* BC18 [AB035551]
	KS4-6	634	421	99	*M. aeruginosa* NIES-843 [AP009552]
	KS4-14	634	421	100	*M. aeruginosa* LMECYA 110
	KS4-19	634	421	99	*M. aeruginosa* LMECYA 142 [EU078501]

a In T-RFLP profiling, the primers used were CYA106f and QCYA792r. For the clone library analysis, the primers used were CYA106f and CYA792r. C.L., clone library
